# A recombinant subunit vaccine for the control of ovine psoroptic mange (sheep scab)

**DOI:** 10.1186/s13567-016-0315-3

**Published:** 2016-02-09

**Authors:** Stewart T. G. Burgess, Francesca Nunn, Mintu Nath, David Frew, Beth Wells, Edward J. Marr, John F. Huntley, Tom N. McNeilly, Alasdair J. Nisbet

**Affiliations:** Moredun Research Institute, Pentlands Science Park, Bush Loan, Edinburgh, Midlothian, Scotland, EH26 0PZ UK; Biomathematics & Statistics Scotland, JCMB, King’s Buildings, Peter Guthrie Tait Road, Edinburgh, Scotland, EH9 3FD UK

## Abstract

Sheep scab, caused by infestation with the mite *Psoroptes ovis*, is highly contagious, causing intense pruritus and represents a major welfare and economic concern. Disease control strategies rely upon chemotherapy, however, sustainability is questionable due to issues of chemical residues, eco-toxicity and acaricide resistance. Control by vaccination is supported by demonstration of protective immunity in sheep previously infested with *P. ovis*. We identified vaccine candidates for *P. ovis* based on: (1) antigens selected by their interaction with host signalling pathways and the host immune-response; and (2) those shown to be either immunogenic or involved in mite feeding. This resulted in the development and validation, in repeated immunisation and challenge trials, of a seven recombinant protein sub-unit cocktail vaccine. Sheep were inoculated on three occasions, 2 weeks apart, along with QuilA adjuvant. Vaccination resulted in highly significant reductions in both lesion size (up to 63%) and mite numbers (up to 56%) following challenge. Mean lesion size in vaccinates was significantly smaller than controls from 1 week post infestation (wpi) until the end of the experiment at 6 wpi. All antigens elicited serum IgG responses following immunisation and prior to infestation, whereas controls did not produce antigen-specific IgG during the pre-infestation period. Vaccinated animals showed an amnestic response, with levels of antigen-specific IgG against muGST, Pso o 1 and Pso o 2 increasing following infestation. This vaccine represents the greatest reduction in lesion size to date with a sheep scab vaccine, providing encouragement for future production of a commercially-viable means of immunoprophylaxis.

## Introduction

Psoroptic mange (sheep scab) caused by infestation with *Psoroptes ovis*, is highly contagious, causes intense pruritus and is a major welfare and economic concern [1, 2]. Currently, disease control relies on chemotherapy; however issues with chemical residues, eco-toxicity and acaricide resistance have raised concerns about the sustainability of this strategy and alternative means of control are desperately needed [3]. The concept of control by vaccination is supported by the demonstration of partial immunity in sheep following previous infestation with *P. ovis* [4–6]: During primary infestation an initial “lag phase”, with small numbers of mites and tight, focal lesions, is followed by a more rapid “growth phase”, with increasing mite numbers and expanding lesions. When this primary infestation is resolved (e.g. by treatment) and sheep are later re-infested, there is an extended lag phase, with lower mite numbers and reduced lesion sizes. Mite-specific IgG responses are similar in primary and secondary infestations but a more rapid induction of mite-specific IgE antibodies occurs in secondary infestations, suggesting that immediate hypersensitivity responses may contribute to immunity [4, 5, 7].

Attempts to vaccinate sheep against *P. ovis* using mite extracts have shown promise, with a 13-fold reduction in mite numbers and >65% reduction in lesion size in vaccinated sheep compared to controls [8]. Similarly, *P. ovis* extracts induce protection against mite challenge in cattle [9]. However, sub-fractionation of these complex extracts failed to identify the protective components involved. Furthermore, the practicality of a vaccine based on native *P. ovis* antigens is limited due to an inability to culture *P. ovis* in vitro, meaning that native antigen extracts would be prohibitively expensive to produce. We have adopted a “rational approach” to recombinant sub-unit vaccine design [10–14] in which host signalling pathways involved in the initial cutaneous pro-inflammatory response to *P. ovis*, upon which the mite relies to initiate its feeding and survival, were elucidated. This allowed identification of mite factors triggering these pathways, which could then be targeted by immunisation thus inhibiting mite survival. As the host:parasite interaction in sheep scab is complex, we hypothesised that incorporating multiple mite antigens into the vaccine, and thus targeting a number of host inflammatory pathways simultaneously, would be most likely to succeed. Similar multiple antigen approaches have been used in the development of effective vaccines against other parasites including the nematodes *Necator americanus* and *Teladorsagia circumcincta* [15, 16].

In this study we employed a recombinant sub-unit cocktail vaccine, using seven *P. ovis* proteins, four of which were identified through the approach described above (Pso o 1; Pso o 2; Pso o 3 and cyclophilin) with three additional antigens identified as either homologues of known allergens (Pso o 10); proteins upregulated during feeding (cathepsin L) or by immuno-screening of *P. ovis* cDNA libraries (mu class glutathione-S-transferase (muGST)) [10–14, 17–22]. Efficacy was tested across repeated trials in sheep using a *P. ovis* challenge model.

## Materials and methods

### Recombinant protein production

The vaccine was composed of seven recombinant proteins as described in Table 1 [14, 18–24]. Pso o 1, Pso o 10, cyclophilin and muGST were soluble in phosphate buffered saline (PBS), whilst Pso o 2, Pso o 3 and Cathepsin L were insoluble. Soluble and insoluble *Escherichia coli*-expressed proteins were induced and purified by nickel-affinity chromatography as described previously [24] and then dialysed against Dialysis Buffer (DB: 20 mM sodium phosphate, 0.5 M NaCl, pH 7.4) containing 2 M urea when purifying insoluble proteins. Protein concentrations were measured using a modified BCA protein assay (Pierce, UK) with BSA standards. Pso o 1 was expressed in *Pichia pastoris*, strain X-33 as described previously [21]. After purification, all antigens were stored at 4 °C, except for Pso o 1, which was stored at −20 °C.

**Table 1:** **Details of the recombinant** ***P. ovis*** **antigens used in the vaccine cocktail**

*P. ovis* antigen	**Accession No.**	**Reference**	**Soluble in PBS** ^**a**^	**Molecular weight (kDa)** ^**b**^	**Expression system**
Cathepsin L	BQ834906.1	[23]	No	25	*E. coli BL21*-*Codon Plus*—pET-22b(+)
muGST	AM991140.1	[14]	Yes	25	*E. coli* *BL21*-*Codon Plus*—pET-22b(+)
Pso o 1	AM269885.1	[21]	Yes	25	*P. pastoris*-*X*-*33*-pPICZαC
Pso o 2	AF187083.1	[24]	No	14	*E. coli* *BL21*-*Codon Plus*—pET-22b(+)
Pso o 3	^c^	^d, e^	No	25	*E. coli* *BL21*-*Codon Plus*—pET-22b(+)
Pso o 10	AM114276.1	[20]	Yes	37	*E. coli* *BL21*-*Codon Plus*—pET-22b(+)
Cyclophilin	AAP03083.1	^d, f^	Yes	38	*E. coli* *BL21*-*Codon Plus*—pET-SUMO

^a^Pso o 1, Pso o 10, cyclophilin and muGST were soluble in PBS, whilst Pso o 2, Pso o 3 and cathepsin L were formulated in Dialysis Buffer (DB).

^b^Predicted molecular weight in kilo Daltons.

^c^Not yet assigned.

^d^Unpublished data.

^e^Pso o 3 identified as a homologue of the house dust mite allergen Der p 3 in an EST from a *P. ovis* cDNA library. The following primer sequences were used to amplify the coding region of Pso o 3, from cDNA derived from mixed stage *P. ovis* as described in [20], downstream of the predicted signal peptide sequence, and to allow subcloning into the expression vector (restriction sites underlined) :Pso o 3-For 5′ GATCCGAATTCGGCATATCGAATGTTTCCACTTCC3′, Pso o 3-Rev-5′ CCGCAAGCTTTACGATTCCGACAATCGTTTTAC3′.

^f^ *P. ovis* cyclophilin identified as an EST from a *P. ovis* cDNA library. The following primer sequences were used to amplify full length *P. ovis* cyclophilin from cDNA derived from mixed stage *P. ovis* as described in [20] : Cyclophilin-For 5′ATGTCAACATGGACCCAAATTCAA′3, Cyclophilin-Rev 5′TTACATTTCACCACATTGTGATATGAT3′. Cyclophilin was subsequently expressed in *E. coli*, confirmed by matrix assisted laser desorption ionisation mass spectroscopy and its peptidyl prolyl *cis*–*trans* isomerase (PPIase) activity confirmed by a coupled enzyme assay as described in [41].

### Immunisation and challenge protocols

#### Trial 1

Thirty sheep scab-naive (animals bred and reared on the MRI farm with no signs of scab observed prior to the start of the study) Texel crossbred lambs (~6 months old) were used in the study as this allowed us to ensure that they had no previous exposure to sheep scab. Lambs were randomly allocated into two equal-sized groups (“vaccine” and “control”) with each group of lambs housed in a separate pen. Lambs in the vaccine group were immunized on three occasions 2 weeks apart with 350 µg of recombinant protein cocktail (50 µg of each of the 7 *P. ovis* antigens) plus QuilA adjuvant (Brenntag Biosector). PBS-soluble proteins were administered together with 5 mg QuilA as a single sub-cutaneous injection into the lateral neck region, whilst insoluble proteins were administered via sub-cutaneous injection into the opposite side of the neck in DB with 5 mg QuilA. Lambs in the adjuvant-only control group were immunized at the same time and via the same routes with equivalent quantities of PBS, DB and QuilA. Two weeks after the final immunisation all lambs were infested, between the withers, with ~50 mixed-stage *P. ovis* mites. Lesion development was assessed weekly and blood samples were collected from all lambs immediately prior to each injection, 1 day pre-infestation and then weekly throughout a six-week infestation period, which is the maximum duration of infestation permitted to remain within the appropriate UK Home Office severity limits. At post mortem (*pm*), 6 weeks post infestation, 3 skin strips (approximately 5 cm × 1 cm) at the leading edge of the lesion were removed from each lamb for enumeration of mites.

#### Trial 2

Trial 2 was similar to Trial 1, with two exceptions: 10 lambs per group were used and 5 skin strips were taken at *pm*. Both trials were performed under the regulations of the UK Animal Procedures Act (1986) and a UK Home Office Project License. Experimental design and statistical power calculations were performed by Biomathematics and Statistics Scotland (BioSS) and were approved by the Moredun Research Institute Experiments and Ethics Committee (Approval Number: Trial 1 = E55/11; Trial 2 = E02/13).

### Assessment of lesion size and mite numbers

The lesion area was measured weekly following infestation by multiplying the length and width of the lesion at the broadest point. Mite numbers were estimated by counting parasites on skin strips from the leading edge of each lesion at *pm* and expressed as mites per cm. An estimate of the total number of mites at the leading edge of the lesion was also determined by multiplying the mite count value by the total lesion perimeter [2 × lesion length (cm) + 2 × lesion width (cm)] for each animal.

### Quantification of antigen-specific IgG

Recombinant antigen-specific IgG levels in serum across the pre- and post-infestation period were assessed for all vaccine antigens by ELISA as described previously [24] with the following exceptions: ELISA for Pso o 3 used a horse-radish peroxidase (HRP)-conjugate of polyclonal antibodies raised in pig against sheep IgG (Dako, UK). ELISA for Pso o 2 was as described in [24] but the antigen was diluted in ddH_2_O rather than carbonate buffer. The responses for each antigen were assessed for each sample in triplicate. OD_450nm_ values were corrected against a reagent blank (no sample control), and all plates incorporated positive (pooled 6 wpi sera) and negative (pre-bleed from sheep scab naïve lambs) serum controls to account for inter- plate variation.

### Statistical analyses

Estimated lesion sizes (cm^2^) were square root transformed and compared using a linear random coefficients model. The model incorporated fixed effects of trial, treatment group, linear and quadratic effects of time (in weeks as a covariate) and a treatment by time interaction, and random effects of intercept and time-specific slope for each lamb. Mite counts, recorded from skin strips from each lamb, were assumed to follow a Poisson distribution and modelled using a generalised linear mixed model (GLMM) with the logarithmic link function. The model included fixed effects of treatment group, trial and a treatment by trial interaction, an offset variable of the logarithm of skin strip length and a random effect of lamb with dispersion parameter estimated to account for the over-dispersion in mite count data. Estimates from this model were used to calculate the total number of mites by combining the estimated lesion perimeters. Mite numbers were not log transformed for statistical analysis, however, to present the data graphically in Figure 2, the log-scale was used to assist data presentation. The data for antibody levels were square root transformed and analysed by an additive linear mixed model, which included fixed effects of treatment, trial and a treatment by trial interaction. Separate smoothing curves were used for the non-linear relationship of the antibody response with time by treatment. The model also incorporated a first-order autoregressive correlation structure between observations at the 13 time points within the same animal and heterogeneity in variance for each trial. All statistical analyses were carried out using R software version 3.0.1 using relevant libraries (base, lme4, mgcv) [25].

## Results

### Lesion size

**Figure 1:**
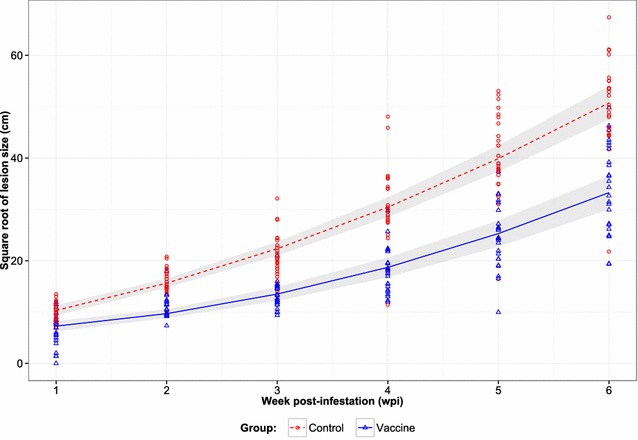
**Lesion development over a 6** **week period post-infestation with** ***P. ovis*** **across repeated vaccine trials.** Lambs were infested with ~50 mites following immunisation with a seven recombinant protein cocktail vaccine with QuilA adjuvant (vaccine) or adjuvant only (control). Data on lesion size are presented on the transformed scale (cm, square root of lesion size). The plot shows observed lesion size of each lamb of vaccine (triangles) and control (circles) groups, estimated mean lesion size of vaccine (solid line) and control (dashed line) groups and corresponding 95% CIs envelop (shaded region).

All lambs across both trials developed a single sheep scab lesion, originating from the site of challenge. Lesion measurements were based on these single and no additional lesions were detected during the infestation period. Figure 1 shows the estimated mean lesion size (transformed data, cm) for vaccine and control groups along with the observed lesion size for each animal at each wpi (across both trials). The mean lesion size, irrespective of treatment groups, did not differ between trials (*p* = 0.641) so the model excluding trial effect is reported. The increase in lesion size with time had a curvilinear relationship (*p* < 0.001). The mean lesion size increased over time for both vaccine and control groups though the rate of increase (cm/wpi) for the control group (8.08 cm ± 0.36) was significantly (*p* < 0.001) higher than in the vaccine group (5.20 cm ± 0.36). Mean lesion sizes (95% lower, upper confidence interval (CI)) in the vaccine and control groups were 52.68 (39.22, 68.13) and 106.46 (86.90, 128.00) cm^2^, respectively at 1 wpi, increasing to 1105.72 (900.77, 1331.64) and 2574.47 (2256.23, 2913.69) cm^2^ for the vaccine and control groups at 6 wpi, respectively. Based on these measurements, lambs in the vaccine group showed, on average, a >57% reduction in lesion size by 6 wpi compared with the control group, with a maximum reduction of 63% in lesion size at 3 wpi.

### Mite numbers

**Figure 2:**
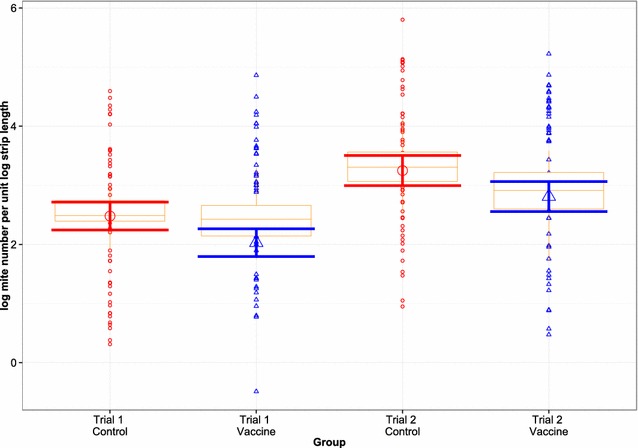
**Mite numbers at the leading edge of the lesion, 6** **weeks post-infestation with** ***P. ovis.*** Data on mite number are presented as the logarithm of mite number per log strip length (cm). The plot shows observed mite number on lambs of vaccine (triangles) and control (circles) groups accompanied with boxplots presenting summary statistics of the observed data, and estimated mean mite number on the log scale (large triangle/circle) and corresponding 95% confidence interval (error bar) for vaccine and control groups during both trials.

The mean mite counts in Trial 2 were significantly higher than in Trial 1 (*p* < 0.001) regardless of treatment group. However, in both trials, the vaccine group had significantly lower mean mite counts compared with the control group (Figure 2, *p* = 0.002). Estimated mean (95% lower, upper CI) mite counts per cm of skin for the vaccine group were 8 (6, 10) and 17 (13, 21) during Trial 1 and 2, respectively. In the control group, estimated mean mite counts were 12 (9, 15) and 26 (20, 34) in the two trials respectively. Accounting for the increased mean lesion perimeter at the leading edge of the lesion for the control group (202.80 cm) compared with the vaccine group (138.56 cm) across both trials, the estimated total mean (95% lower and upper CI) mite numbers for the vaccine and control groups for Trial 1 were 1055 (835, 1333) and 2414 (1915, 3048), respectively. Corresponding estimates for Trial 2 were 2292 (1767, 2976) and 5251 (4045, 6811), respectively. Across both trials the vaccinated lambs on average had a >56% reduction in total mite numbers at the leading edge of the lesion compared with the control group.

### Serum IgG responses to recombinant *P. ovis* antigens

**Figure 3:**
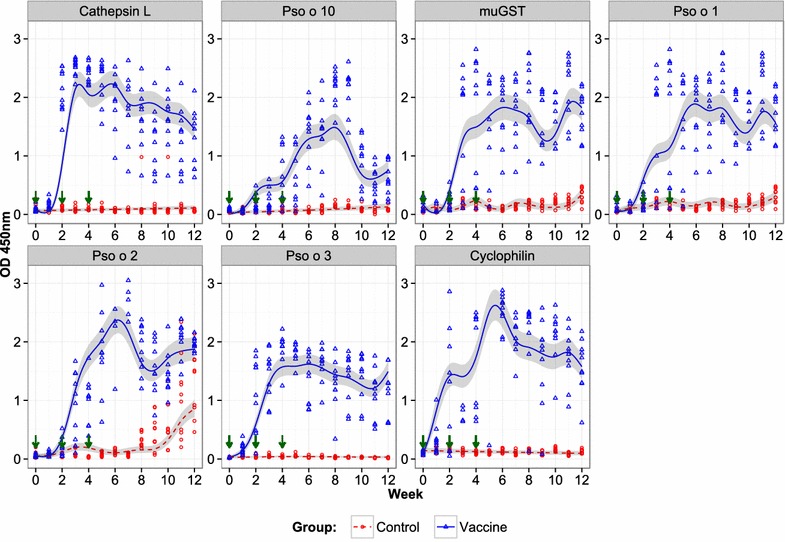
**Antigen-specific antibody (IgG) levels in serum over a 6** **week period post-infestation with** ***P. ovis.*** Serum IgG responses specific for cathepsin L; Pso o 10; muGST; Pso o 1; Pso o 2, Pso o 3 and cyclophilin, respectively over a 6 week period of infestation with *P. ovis* during Trial 2 only (2013). Data on IgG levels are presented on the observed scale (OD_450nm_). The plot shows observed IgG levels of each lamb of the vaccine (triangles) and control (circles) groups, estimated mean IgG level of vaccine (solid line) and control (dashed line) groups and corresponding 95% confidence interval (CI) envelope (shaded region). Green arrows indicate timing of immunisations.

The antibody responses to all seven recombinant antigens for vaccine and control groups in Trial 2 are shown in Figure 3. All vaccinated animals generated an IgG antibody response to the seven antigens. The vaccine group had statistically significantly higher antibody levels to all antigens compared with the control group during both pre- and post-infestation periods. IgG levels for most vaccine antigens peaked at 7–14 days after the final immunisation and then declined. By 4 wpi, increased antigen-specific serum IgG levels were observed for the muGST, Pso o 1 and Pso o 2 indicating a potential amnestic response to these antigens. Serum from control animals had no measurable vaccine antigen-specific IgG prior to infestation, but did contain Pso o 2-specific IgG by 3 wpi and Pso o 1- and muGST-specific IgG by 6 wpi demonstrating that these antigens were recognised by the host immune response during exposure to *P. ovis* mites, following infestation.

## Discussion

The data presented here demonstrate the efficacy of a recombinant subunit sheep scab vaccine based on a cocktail of seven *P. ovis* antigens. When administered to lambs, the vaccine resulted in highly significant reductions in both lesion size (57%) and mite numbers (56%) following challenge in repeated protection trials. The lesions in the immunised lambs were significantly smaller than in matched control animals from 1 wpi until the end of the experiment at 6 wpi. In sheep scab, disease is transmitted via direct contact or fomites, so even modest decreases in lesion size and the concomitant reductions in mite numbers may limit disease spread [26–29]. All vaccine antigens elicited serum IgG responses following immunisation whereas animals in the control group did not possess antigen-specific IgG during the pre-infestation period. In addition, an amnestic response was observed in vaccinated animals, following mite challenge, with levels of antigen-specific IgG against muGST, Pso o 1 and Pso o 2 increasing following infestation with *P. ovis*. Serum IgG antibodies which bound recombinant Pso o 2 were demonstrated in the infested control animals, underpinning its use as a diagnostic antigen for the detection of sheep scab. In contrast no antigen-specific IgG response was observed for the mite antigens, Pso o 3 and Pso o 10. A previous study by our group demonstrated a similar lack of specific IgG reactivity to Pso o 10 in sheep undergoing a primary infestation with sheep scab and as such we would not expect to see an IgG response in these animals to Pso o 10 [20]. Detection of IgG to a defined antigen depends on many factors, including the amount of antigen that the host is exposed to and the relative immunogenicity of the antigen. It should also be noted that the Pso o 3 and Pso o 10 used for the ELISAs in this study were bacterial recombinant antigens, which may have structural differences to the native antigens and thus be poorly recognized by the mite-induced IgG response. The magnitude of the experimental challenge in the model described here is likely to be much greater than in a field outbreak, as the numbers of mites used (~50 mites per lamb) are likely to be substantially higher than those experienced during a natural infestation where only small numbers of mites, or even a single ovigerous individual, may be sufficient to establish a lesion [30–32]. Additionally, as a result of the potentially limited numbers of mites encountered in a natural challenge, field infestations may develop more slowly over a longer period of time encompassing several months rather than the 6 weeks described here [30]. Based on the estimates of slopes in conjunction with the line plots of lesion size for both vaccine and control groups (Figure 1) it may be inferred that the lesion size would increase progressively with time, beyond the period investigated in the current experiment, and hence, the difference in lesion size between control and vaccine groups could become more pronounced at later time points. Therefore, the ultimate efficacy of this vaccine may actually be greater than demonstrated here; however, further studies under field conditions are required to validate this hypothesis.

The subunit vaccine described here represents the greatest reduction in lesion size with a recombinant sheep scab vaccine to date, providing encouragement for future production of a commercially-viable means of immunoprophylaxis. Previous attempts have been made to produce an effective vaccine for sheep scab. For example, Nisbet et al. [14] produced a multi-protein recombinant vaccine based on *P. ovis* allergens, however the efficacy of this vaccine could not be determined due to the high degree of variability in the lesion size and mite numbers in the controls. Other efforts have focused on the use of native extracts of *P. ovis* to generate protective immunity: a vaccine based on *P. ovis* soluble proteins was previously tested in cattle, with 8/14 vaccinated calves being free of palpable lesions by 8 wpi compared to 3/14 in the controls [9]. Unfortunately, native extract based vaccines are unlikely to be commercially feasible due to the absence of in vitro culture systems for *P. ovis* to supply sufficient material for commercial production and also the lack of reproducibility with which these extracts can be produced. The use of a recombinant cocktail vaccine is, therefore, likely to be required for controlling complex eukaryotic parasites and may have advantages over single protein vaccines [33]. Whereas single point mutations in drug targets can lead to drug resistance, this is far less likely in a vaccine relying on multiple B cell epitopes present in a cocktail vaccine [28, 33].

Mathematical modelling has demonstrated that vaccines to control endoparasites may not need to achieve the same degree of efficacy as chemotherapeutics to achieve economic control of parasites, however, as these models were based on gastrointestinal nematode infections they may not be directly applicable to psoroptic mange [34–36]. Sterile immunity against many ectoparasites may not be achievable via vaccination and, unlike chemotherapeutics, an ectoparasite vaccine may not induce a rapid knockdown of parasite population nor necessarily protect individuals from being parasitized [29, 36]. Vaccination does have the potential to provide greater protection from re-infestation than achievable with chemotherapeutic control, which currently ranges from low levels of protection with a single dose of doramectin and up to 60 days for moxidectin in a long acting formulation. If used as part of an integrated control program, vaccines may reduce parasite populations over successive generations and, in the short term may mitigate the effects of parasitism by controlling population growth, limiting clinical pathology and alleviating the more extreme welfare symptoms [29]. Furthermore, vaccination may also reduce disease impact by blocking or reducing the spread of disease within and between flocks [29], although this is yet to be formally tested.

Given the likely efficacy achievable through vaccination, vaccines should not be considered as a single control measure for sheep scab but rather as an additional arm in a growing arsenal of tools available for coordinated control, including diagnostic tests, existing chemotherapeutics and effective biosecurity. However, to encourage producers to begin to switch from their current reliance on chemotherapeutics to a more coordinated approach involving anti-parasite vaccines, these products will have to demonstrate clear benefits, i.e. be efficacious, cost effective, environmentally friendly and sustainable [37]. One potential disadvantage of the cocktail approach is the additional costs involved in commercial production of a vaccine based on multiple antigens and, although not necessarily a barrier to commercial success, it is important to ensure that costs are reflective of the market. This may require further distillation of antigens required for protection, or formulation of protective antigens and/or epitopes within a single fusion protein, as recently demonstrated with an *E. coli* O157:H7 subunit vaccine [38] and also by co-expression of multiple copies of a rabies virus glycoprotein using a foot and mouth disease virus expression system incorporating the 2A peptide [39]. It is also critical at this stage to develop effective strategies to use this vaccine in the field. This will require identifying the optimal methods of integrating the vaccine with existing controls. For example this may involve combined use of diagnostic tests [24, 40] to identify and confirm outbreaks of disease, treatment with existing chemotherapeutic compounds and administration of vaccine to contiguous properties to limit further transmission.
